# Supplementary material for: Resolving the full spectrum of human genome variation using Linked-Reads
Source: Genome Res. 2019 Apr;29(4):635–45. doi: 10.1101/gr.234443.118 (PMC6442396; doi:10.1101/gr.234443.118)
Supplement: Supplemental Material [file supp_gr.234443.118_Supplemental_longranger-2.2.zip › longranger-2.2.tar.bz2!/lr2.2-public/lib/stan/cmdstan/stan/doc/api/html/math_2matrix_2log__softmax_8hpp_source.html]

Stan: src/stan/math/matrix/log\_softmax.hpp Source File


|  |  |
| --- | --- |
|  | Stan  2.5.0  probability, sampling & optimization |


- Main Page
- Related Pages
- Namespaces
- Classes
- Files

- File List
- File Members

All Classes Namespaces Files Functions Variables Typedefs Enumerations Enumerator Friends Macros Pages

- src
- stan
- math
- matrix

log\_softmax.hpp

Go to the documentation of this file.

1 #ifndef STAN\_\_MATH\_\_MATRIX\_\_LOG\_SOFTMAX\_HPP

2 #define STAN\_\_MATH\_\_MATRIX\_\_LOG\_SOFTMAX\_HPP

3

4 #include <cmath>

5 #include <sstream>

6 #include <stdexcept>

7 #include <stan/math/matrix/Eigen.hpp>

8 #include <stan/math/matrix/log\_sum\_exp.hpp>

9 #include <stan/math/error\_handling/matrix/check\_nonzero\_size.hpp>

10

11 namespace stan {

12  namespace math {

13

42  template <typename T>

43  inline Eigen::Matrix<T,Eigen::Dynamic,1>

44  log\_softmax(const Eigen::Matrix<T,Eigen::Dynamic,1>& v) {

45  using std::exp;

46  using std::log;

47  using stan::math::log\_sum\_exp;

48  stan::math::check\_nonzero\_size("log\_softmax(%1%)",v,"v", (double\*)0);

49  Eigen::Matrix<T,Eigen::Dynamic,1> theta(v.size());

50  T z = log\_sum\_exp(v);

51  for (int i = 0; i < v.size(); ++i)

52  theta(i) = v(i) - z;

53  return theta;

54  // T sum(0.0);

55  // T max\_v = v.maxCoeff();

56  // for (int i = 0; i < v.size(); ++i)

57  // sum += exp(v(i) - max\_v); // log\_sum\_exp trick

58  // T log\_sum = log(sum);

59  // for (int i = 0; i < v.size(); ++i)

60  // theta(i) = (v(i) - max\_v) - log\_sum;

61  // return theta;

62  }

63

64  }

65 }

66 #endif

stan::math::log\_sum\_exp

boost::math::tools::promote\_args< T1, T2 >::type log\_sum\_exp(const T2 &a, const T1 &b)

Calculates the log sum of exponetials without overflow.

**Definition:** log\_sum\_exp.hpp:49

Eigen.hpp

stan::math::log\_softmax

Eigen::Matrix< T, Eigen::Dynamic, 1 > log\_softmax(const Eigen::Matrix< T, Eigen::Dynamic, 1 > &v)

Return the natural logarithm of the softmax of the specified vector.

**Definition:** log\_softmax.hpp:44

log\_sum\_exp.hpp

check\_nonzero\_size.hpp

stan::agrad::log

fvar< T > log(const fvar< T > &x)

**Definition:** log.hpp:15

stan::agrad::exp

fvar< T > exp(const fvar< T > &x)

**Definition:** exp.hpp:16

stan::math::check\_nonzero\_size

bool check\_nonzero\_size(const char \*function, const T\_y &y, const char \*name, T\_result \*result)

Return true if the specified matrix/vector is of non-zero size.

**Definition:** check\_nonzero\_size.hpp:33


---

[ Stan Home Page ]

*© 2011–2014,
Stan Development Team.*
